# Adaptation of a digital health intervention for rural adults: application of the Framework for Reporting Adaptations and Modifications-Enhanced

**DOI:** 10.3389/fdgth.2025.1493814

**Published:** 2025-02-18

**Authors:** Maura M. Kepper, Callie Walsh-Bailey, Loni Parrish, Ainsley Mackenzie, Lisa M. Klesges, Peg Allen, Kia L. Davis, Randi Foraker, Ross C. Brownson

**Affiliations:** ^1^ Prevention Research Center, Brown School, Washington University in St. Louis, St. Louis, MO, United States; ^2^ Department of Medical Social Sciences, Northwestern University Feinberg School of Medicine, Chicago, IL, United States; ^3^ Department of Surgery, Washington University School of Medicine, St. Louis, MO, United States; ^4^ Institute for Informatics, Washington University School of Medicine, St. Louis, MO, United States; ^5^ Division of General Medical Sciences, Department of Medicine, Washington University School of Medicine, St. Louis, MO, United States; ^6^ Siteman Cancer Center and Division of Public Health Sciences, Department of Surgery, Washington University School of Medicine, St. Louis, MO, United States

**Keywords:** digital health, adaptation, implementation science, chronic disease prevention, rural health

## Abstract

**Introduction:**

Adaptation is a key aspect of implementation science; interventions frequently need adaptation to better fit their delivery contexts and intended users and recipients. As digital health interventions are rapidly developed and expanded, it is important to understand how such interventions are modified. This paper details the process of engaging end-users in adapting the PREVENT digital health intervention for rural adults and systematically reporting adaptations using the Framework for Reporting Adaptations and Modifications-Enhanced (FRAME). The secondary objective was to tailor FRAME for digital health interventions and to document potential implications for equity.

**Methods:**

PREVENT's adaptations were informed by two pilot feasibility trials and a planning grant which included advisory boards, direct clinic observations, and qualitative interviews with patients, caregivers, and healthcare team members. Adaptations were catalogued in an Excel tracker, including a brief description of the change. Pilot coding was conducted on a subset of adaptations to revise the FRAME codebook and generate consensus. We used a directed content analysis approach and conducted a secondary data analysis to apply the revised FRAME to all adaptations made to PREVENT (*n* = 20).

**Results:**

All but one adaptation was planned, most were reactive (versus proactive), and all adaptations preserved fidelity to PREVENT. Adaptations were made to content and features of the PREVENT tool and may have positive implications for equity that will be tested in future trials.

**Conclusion:**

Engaging rural partners to adapt our digital health tool prior to implementation with rural adults was critical to meet the unique needs of rural, low-income adult patients, fit the rural clinical care settings, and increase the likelihood of generating the intended impact among this patient population. The digital health expansion of FRAME can be applied prospectively or retrospectively by researchers and practitioners to plan, understand, and characterize digital health adaptations. This can aid intervention design, scale up, and evaluation in the rapidly expanding area of digital health.

## 1 Introduction

Cardiovascular disease (CVD) is the leading cause of death in the United States (US), and disproportionately affects the 60 million people living in rural areas (1, 2). CVD prevalence is 40% higher among rural than urban residents (1), and while CVD mortality decreased by 6.4% in urban areas between 2010 and 2022, CVD-related deaths increased nearly 1% in rural areas over the same time period (3). The American Heart Association's (AHA's) Life's Essential 8 has identified modifiable health behaviors (i.e., physical activity and food intake) as critical for cardiovascular health and prevention of CVD (4). Evidence-based interventions that improve physical activity, food intake, and body mass index (BMI), can prevent up to 40% of deaths (5). Yet, only 20% of US adults meet healthy lifestyle recommendations and those in rural, low-income areas are particularly challenged by unmet social needs (e.g., food insecurity, lack of transportation) (6–12).

Promotion of healthy behavior is impacted by the conditions in which people live and work (4). Emphasized in the Chronic Care Model (13), connecting patients with health-promoting resources, e.g., healthy lifestyle programs, social services, and food resources, is critical to create opportunities for individuals to improve health behaviors and attain their highest level of health, particularly in rural communities (14). Rural communities face unique challenges to healthy behaviors, such as the cost and availability of nutritious foods and low walkability (15, 16). Additionally, minoritized racial/ethnic groups and low socioeconomic status populations often encounter similar barriers, further complicating their efforts to maintain a healthy lifestyle (17). Digital tools can be utilized to gather additonal health behavior and social needs data that is not typically included in the electronic health records. The digital health tool data is used to promote data-informed, individually tailored care within routine clinical encounters (18). When using digital interventions in rural communities, considerations of digital literacy and the potential for limited access to and low-quality of broadband are important (19–21).

To address these challenges in promoting cardiovascular health, our team developed PREVENT, a digital health tool intended for healthcare providers to use during routine clinical encounters to provide data-informed cardiovascular health education, individualized evidence-based physical activity and food intake goals, and resources to promote health behavior change among patients with overweight/obesity (22–24). PREVENT was originally developed for adolescents with overweight/obesity and piloted with healthcare providers in urban clinical settings (22). To be effective among rural adults with overweight/obesity, PREVENT must be adapted to meet the unique needs of adults, rural communities, and their clinical care settings. Evidence-based interventions are frequently modified using intentional adaptations, i.e., planned or purposeful changes to the design or delivery of the intervention, and unanticipated changes during the testing or implementation process. Modifications may facilitate successful implementation and sustainability by improving the fit between interventions and the target population or the routine delivery context, yet some modifications may deviate from the intended intervention appraoch (25). Further, the potential positive or negative implications such adaptations and modifications have on equity are not well documented or understood. Utilizing a systematic tracking process, such as the Framework for Reporting Adaptations and Modifications to Evidence-Based Interventions (FRAME), facilitates understanding of how and to what degree the adaptation affects implementation and effectiveness (26, 27). This approach also fosters consistency and efficiency for the development of future programs' implementation planning by providing detailed explanations and transparency in decision making.

Given the unique challenges of using digital health for health promotion in rural settings, it is important to utilize a systematic, user-engaged approach to proactively optimize the intervention while maintaining fidelity with the program's goal (28). The users and the community of interest are best suited to inform adaptations as they are most knowledgeable about their needs, preferences, organizational capacity (resources and staffing), and implementation challenges (29–31).

The primary objective of this paper is to demonstrate the process of engaging end-users in adapting PREVENT for rural adults and systematically reporting adaptations using FRAME. The secondary objective was to tailor FRAME for digital health adaptation tracking and to document potential implications for equity.

## 2 Materials and methods

### 2.1 PREVENT tool

The original development of the PREVENT digital health tool is described in detail by Kepper and colleagues (22). PREVENT is a digital clinical support tool used by healthcare providers to deliver health behavior counseling to patients during their clinical care visit, and follow-up with them after the visit to support health behavior change and promote cardiovascular health. The original version of the PREVENT tool was designed for adolescents (≤18 years of age) and used the American Heart Association (AHA's) Life's Simple 7 cardiovascular health indicators and algorithm to categorize patient health behaviors (food intake, physical activity, smoking) and clinical indicators (BMI, blood pressure, blood glucose, cholesterol) into ideal, intermediate, or poor ranges. PREVENT integrates these data into an interactive dashboard that summarizes the patient's cardiovascular health status. Based on a patient's current behaviors and health status, the PREVENT tool generates personalized, evidence-based food intake and physical activity goals. The tool includes a community resource map cataloging resources to support healthy eating and physical activity (e.g., farmer's markets, food pantries, community centers, parks and playgrounds). PREVENT summarizes this information into a patient prescription that is delivered electronically via text or email, per patient communication preferences. Additionally, PREVENT sends monthly automated goal check-ins to patients to report their progress, troubleshoot barriers, and provides tailored encouragement messages and new goals.

To effectively use PREVENT, healthcare teams are trained on how to use the tool (e.g., how to locate a patient profile, how to use the tool's features) and employ shared-decision-making techniques to prioritize behavior change goals with the patient. In two pilot feasibility trials, the PREVENT tool was tested in multiple care delivery models to align with existing practice workflows, including by a single provider or using a team-based approach involing two roles on the care team to address different aspects of the tool (one provider offers cardiovascular health education, another addresses behavior changes goals and resources) (23, 24). PREVENT was orinigally tested as a standalone, Health Insurance Portability and Accountability Act (HIPAA) compliant webtool, and can also be integrated into various electronic health record systems. To ensure the tool facilitates personalized counseling, it administers surveys to the patient and relies on self-report and objective patient data that is within the patient's chart (e.g., address, weight, height), which can be manually entered or automatically pulled when the tool is integrated into the electronic health record.

### 2.2 PREVENT trials and adaptation data sources

Changes to the PREVENT tool, reported in the results section of this paper, were informed by an array of data sources, implementers, and recipients. Adaptation of the PREVENT tool took place in iterative phases over the course of the three projects described below. Table 1 summarizes the information sources informing the adaptation of the PREVENT tool. All projects received Institutional Review Board approval (#202004230, #202007026, #202209074, #202211172) and followed appropriate consent procedures for all participants.

**Table 1:** Data sources informing adaptations.

Data Source	Data type	Participant(s)	Time period
Pilot Trials			
Interviews with users	Qualitative	Heatlhcare team members who implemented the tool & patient participants in pilot trials	Late 2021 – early 2022
Direct observation of use	Qualitative, quantitative	Heatlhcare team members who implemented the tool & patient participants in pilot trials	Mid-late 2021
Informal feedback during pilot trials	Qualitative	Implementing clinicians	Mid 2021 – early 2022
Pilot trial advisory board	Qualitative	Researchers, clinicians, health system leaders, community organizations, patients	Early 2021 – Mid 2022
Post-trial debrief meetings	Qualitative, quantitative	Heatlhcare team members who implemented the tool	Early-mid 2022
Rural planning project			
Interviews with prospective users	Qualitative	Physicians, nurse practitioners, nurses, and community health workers in rural clinics who may implement the tool in the future	Early 2023
Rapid clinic ethnography	Qualitative, quantitative	Rural clinic teams (healthcare teams, staff, clinic managers)	Early 2023
Rural advisory board	Qualitative	Researchers, healthcare team members, health system leaders, community organizations, patients	Early 2023

We conducted two randomized pilot feasibility trials of the PREVENT tool among two patient populations receiving care in clinics affiliated with a large academic medical center. The first trial among adolescent patients with obesity aged 12–18 receiving care in a multi-disciplinary weight management clinic (referred to as “Healthy Start” throughout), launched in early 2021 (32). The second trial conducted later in 2021 was among adolescent and young adult (AYA) cancer survivors aged 12–39 receiving comprehensive survivorship care across three clinics in an urban metro area (referred to as “AYA cancer survivorship” throughout) (24). The study design was similar across both trials, which included patient-level randomization to an intervention condition in which their provider used the PREVENT tool during their routine clinic visit or a usual care control condition. In both trials, we observed a subset of intervention visits to assess PREVENT's use and fit with workflow. We conducted post-intervention surveys and semi-structured interviews with healthcare providers who delivered the tool and with patients and parents of minor patients who received PREVENT. The research team also received informal feedback from healthcare team members during these trials while in the clinic conducting recruitment and providing technical assistance. Informal feedback was documented in field notes as barriers and facilitators in real-time by the research team. At the end of each trial, the research team conducted one hour virtual debrief meetings with the healthcare teams from the two trials. These meetings included participant validation of key learnings from the trials, collection of quantitative ratings to prioritize barriers and facilitators to implementation, and recommended implementation strategies and adaptations to support PREVENT's integration into routine practice.

In addition to the two pilot feasibility studies, we conducted a one-year planning project (referred to as “rural planning project” throughout) to adapt PREVENT for use via a team-based care approach for adult patients in rural clinics, in preparation for a larger scale hybrid implementation-effectiveness trial. This rural planning project included rapid clinic ethography (33), consisting of site visits to observe clinic workflows, document review (e.g., local resource binders), and semi-structured qualitative interviews. Informal conversations during site visits between the research team and clinic managers, staff, and healthcare team members, were documented in field notes from the visit. Semi-structured qualitative interviews with prospective users of the PREVENT tool [e.g., physicians, nurse practitioners, social workers, community health workers (CHWs)] were audio-recorded and transcribed. The interview guide is included as Supplementary Data Sheet 1.

All three studies included an advisory board (composition varied by project) comprised of researchers, clinicians, health system leaders (e.g, chief executive officer, medical director, information technology director), community organization partners (e.g., rural community resource council), and patients; suggested adaptations were documented from advisory board meeting notes. Finally, the PREVENT study's principal investigator (PI, MMK) had regular meetings with the software development team who conducted the programming work to develop PREVENT. The research team kept notes and an Excel tracker of all changes made to the PREVENT tool since its original development.

### 2.3 PREVENT adaptation coding

#### 2.3.1 Codebook development

Given the novelty of digital health interventions in the field of implementation science, our team modified FRAME to improve its applicability to characterize changes to the PREVENT tool. Refinements to existing elements of FRAME included modifying code labels to better fit the context of the PREVENT studies (e.g., changing “treatment/intervention team” to “care team”), adding new coding options within a category (e.g., addition of “feature” as an option within “what is modified”), and operationalizing code definitions to fit the digital context.

**Figure 1:**
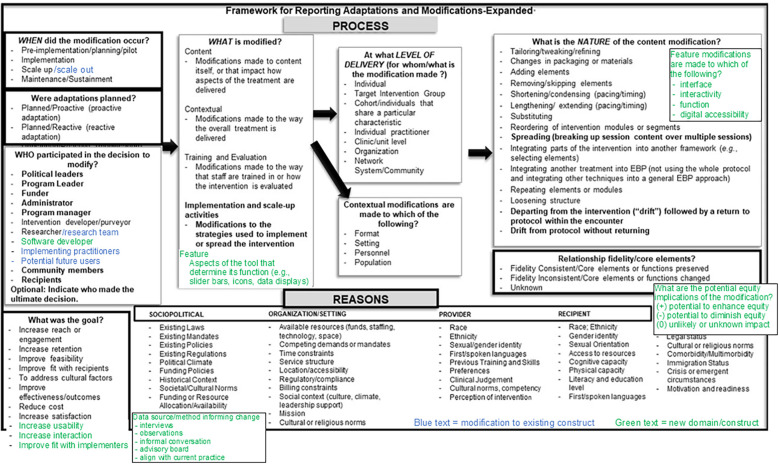
Frame figure.

Additionally, we added new elements to FRAME on end-user participation in adaptations and equity implications. New elements included: (1) “source/method informing change” to capture the participant type and data source from which information informing the change was derived; (2) options to classify changes made to the features of a digital health tool; and (3) a component to code the positive and negative equity implications of each adaptation (Figure 1; Supplementary Table 1). The coding for equity implications was guided by an approach developed by the second author, and was broadly inclusive of potential to impact equitable implementation processes (e.g., fair distribution of decision-making power among healthcare team roles) and outcomes (e.g., equitable reach of the intervention to marginalized patient groups).

#### 2.3.2 Coding

The research team tracked and systematically coded adaptations made to the PREVENT tool across the three aforementioned studies. Qualitative data from the PREVENT studies were analyzed using a directed content analysis approach (34, 35), and we conducted secondary data analysis of the coding memos to ascertain the modifications made to the PREVENT tool or suggestions from which modification decisions were derived. Adaptations were categorized based on FRAME (26). This commonly used implementation science framework includes detailed elements for categorizing the types of modifications made to an intervention (see Supplementary Data Sheet 1 for detailed codebook).

The study PI and three research assistants assembled a list of adaptations from the data sources described above, entering a brief narrative summary of each change into the Excel tracker. The team then pilot coded a subset of the adaptations using the revised FRAME codebook. The team reviewed the coding, provided comments and edits asynchronously, then met to generate consensus and revise the codebook. The team then independently coded remaining adaptations, repeating the process of asynchronous review by an independent coder and consensus discussion to resolve disagreements.

## 3 Results

**Figure 2:**
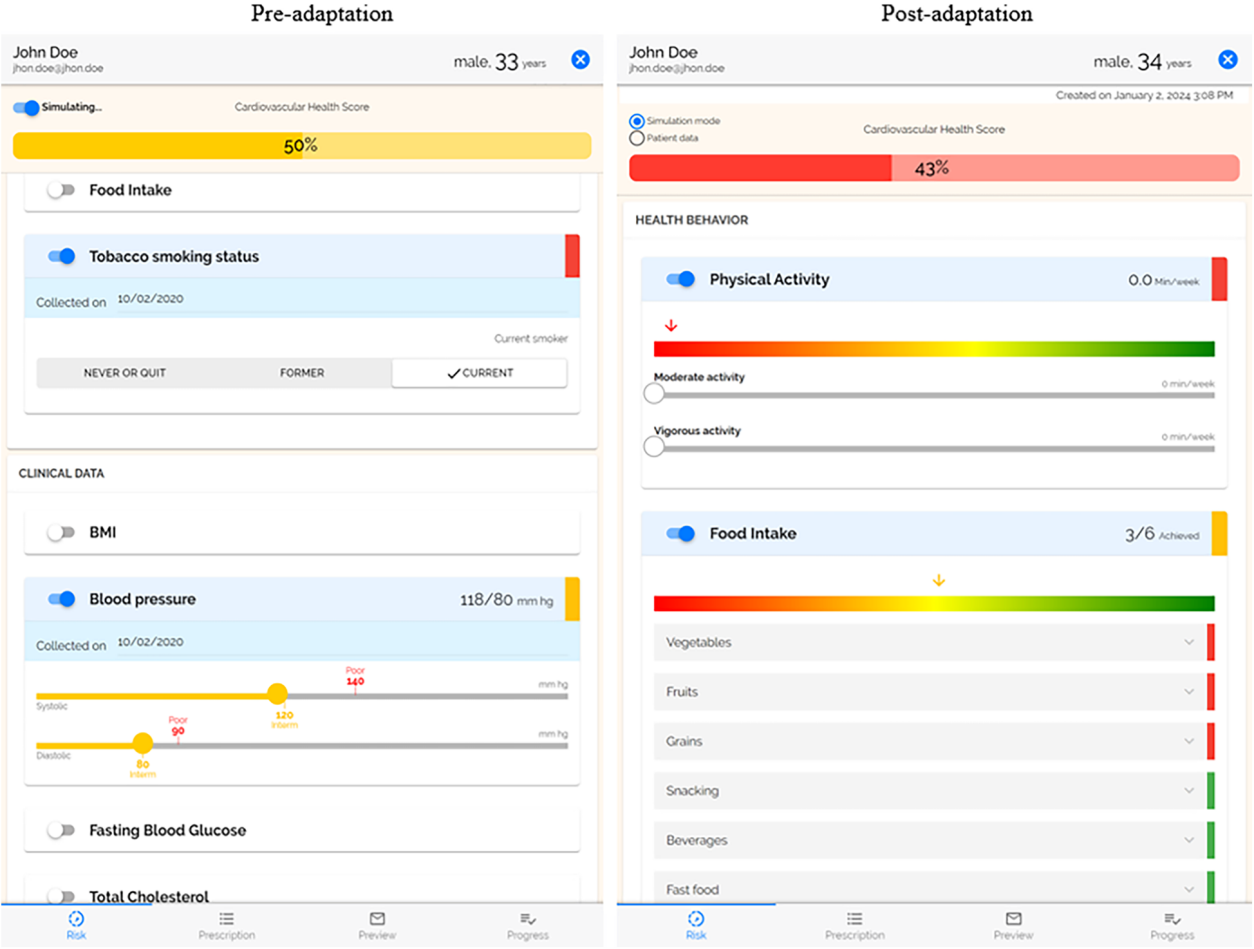
Side by side of risk profile. The post-adaptation risk profile uses the AHA's Life's Essential 8, which added sleep and updated the scoring. Physical activity and food intake were moved to the top as the focus of the PREVENT intervention.

A summary of coded adaptations is presented in Table 2 (Supplementary Table 2 provides the expanded table of adaptations with all FRAME coding). All adaptations were fidelity consistent and preserved the core functions of the original PREVENT tool. Prior to the second trial of PREVENT in AYA cancer survivors (12–39 years), PREVENT was proactively expanded for adults (>18 years of age) based on input from our advisory board to increase the reach and equity of PREVENT, improve the fit, and increase the effectiveness among this age group. This expansion required changes to the content (e.g., education materials, goals), features (e.g., cardiovascular health scoring) and context (adult clinics). The reason for this change was preferences by clinicians who felt this tool would benefit adult patients and was influenced by funding support for behavior change interventions in AYA cancer survivors who are at an increased risk for poor cardiovascular health compared to their counterparts. Another major adaptation of PREVENT occurred following the pilot trials with the release of the AHA's Life's Essential 8 (34) (an update to Life's Simple 7), which added sleep as a risk factor and updated the way in which cardiovascular health was scored (Figure 2). To align with this new guidance, we updated features and content of our tool. Specifically, surveys were updated to include sleep, add necessary questions on medication use for blood pressure and cholesterol, and change food intake questions to align with the Mediterranean Eating Pattern for Americans (MEPA) screener (36) that was recommended by Life's Essential 8. The risk calculation was updated based AHA's changes, and our educational content was updated with the goal of improving the effectiveness and outcomes of our tool. The addition of sleep may increase patients' knowledge of the importance of sleep and have positive equity implications as minoritized racial/ethnic groups and low socioeconomic status populations have higher prevalence of sleep deficiency (37, 38).

**Table 2:** Summary table of coded adaptations to the PREVENT tool area of change.

	Description of change	Source	What was changed?	What was modified?	Potential equity implications
Changes during pilot trial implementation					
Expansion to adults (>18 years of age)	Updated cardiovascular health cut points and scoring; updated behavior change goals and educational materials	Pilot trial advisory board	increase reach or engagement; improve fit with recipients; improve effectiveness/outcomes	content, feature	(+) expand reach to other populations (adults) who may benefit from PREVENT
Patient centered/personalization	Ability for care team to edit (toggle goal on/off, add free text) the goals.	Interviews with users; pilot trial advisory board	Improve fit with recipients; increase interaction; increase satisfaction of implementer; increase satisfaction among recipient	content, feature	(+) ability to tailor goal to patient needs/preferences and focus on most achievable or desirable starting place; (−) potential for provider bias to influence whether patient receives goal information
	Displayed patient's motivation to change their physical activity and food intake behaviors.	Interviews with users	Improve fit with recipients	content	(+) ability to tailor goal to patient needs/preferences and focus on most achievable or desirable starting place; (−) potential for provider bias to influence whether patient receives goal information
Patient engagement	Moved activity and diet to the top of the risk profile	Interviews with users; informal feedback during pilot trials	increase usability	feature	(+) De-emphasized focus on body mass index/weight (previously at the top of the risk profile) to avoid stigma/bias; (0) potential remains for use of stigmatizing language to discuss behaviors or weight, but did not increase potential of bias
Resource support for rural communities	Added digital resource repository	Interviews with users; informal feedback during pilot trials; direct observations of use	Improve fit with recipients, To address cultural factors, Improve effectiveness/outcomes	content, feature	(+) increase availability of resources for patients without reliable transportation; may increase availability of free/low cost resources (−) access limited for patients with low digital literacy and those without internet access and/or electronic device
Workflow improvement	Defaulted the risk profile to simulation mode; button to change from simulation mode to patient data mode clarified	Interviews with users; informal feedback during pilot trials; direct observations of use	increase usability, improve fit with implementers	feature	(0) Unknown/unlikely impact on equity
	Improve refresh capability to fix issue with simultaneous users	direct observations of use; informal feedback during pilot trials	feasibility; increase usability; increase satisfaction of implementer	feature	(+) increase ability and efficiently for multiple care team members, including CHWs, to assist/work with the patient
Changes during scale out to rural settings					
Alignment with AHA's life's essential 8	Added to sleep risk profile, updated surveys; changed risk calculation	American Heart Association guidance	Improve effectiveness/outcomes	content, feature	(+) Minoritized racial/ethnic groups and low SES populations have higher prevalence of sleep deficiency; potential to improve education, awareness about sleep among marginalized populations
Patient centered/personalization	Food goals tailored to patient's current behavior.	Interviews with prospective users; direct observations of use	improve fit with recipients, improve effectiveness/outcomes	content	(+) ability to deliver a goal that tailored to the patients current behaviors to focus on the most achievable or desirable staring place; (−) potential for provider bias to influence whether patient receives goal information
	Modifications to patient demographic info (change sex to sex at birth, add option for gender to include additional identities beyond m/f)	Direct observation of use; interviews with prospective users	increase feasibility; improve fit w recipients; address cultural factors	content, feature	(+) improves ability for provider to address patients in their preferred way, build rapport/trust; (−) potential for provider bias based on gender identity; potential to offend a patient who does not think gender identity differs from biological sex
Patient engagement	Improved prescription (added educational content, added resource information, enlarged text, updated graphics)	Interviews with prospetive users; rapid clinic ethnography	increase usability, retention, improve fit with recipients	content	(+) improves literacy level of content to make understandable for all; adds educational information to support those less knowledgeable about health behaviors and cardiovascular health; provides more information on resources to support individuals who may not be able to access the internet or a computer to look up this information on their own; adds larger font to reduce difficulty reading smaller font sizes; (−) content not available in a language other than English; written material may not be sufficient illiterate populations
	Changed follow-up surveys and tracking (chart organizing survey responses)	Interviews with prospective users; rapid clinic ethnography; rural advisory board	retention; increase satisfaction of implementer	content, feature	(0) Unknown/unlikely impact on equity
Resource support for rural communities	Added patient's preferences for resources to surveys and automatically select preferred resources within a.5 mile radius of their home. If <5 resources in that area, then zoom out until at least 5 are selected.	Interviews with prospective users; direct observations of use	improve fit with implementers; increase satisfaction of recipients; increase usability	feature	(+) improves awareness of local resources; increases selection of resources in closest proximity to patient home, which may improve accessibility for patients with limited/no transportation (−) Resource access may not be equitable for patients without quality, affordable resources nearby and who lack transportation
	Added social needs resources	Interviews with prospective users; rapid clinic ethnography; rural advisory board	Improve effectiveness/outcomes, increase satisfaction of implementer, increase satisfaction with recipients, improve fit with implementers, to address cultural factors	content, feature	(+) Minoritized racial/ethnic groups and low SES populations have higher prevalence social needs that impact their health; improve efficiency of resource delivery for CHWs by consolidating and sharing information about resources across the care team
	Added date last updated and a verified check mark for resources that were manually entered	rapid clinic ethnography; rural advisory board	Improve feasibility, Improve fit with recipients; improve fit with implementer; address cultural factors; increase satisfaction of implementer	feature	(+) resources in under-resourced settings are frequently changing, the addition of this feature ensures that patients receive resources that still exist and fit their needs.
	Added detailed information & functionality (cost, eligibility, criteria, and services provided) for each resource	Interviews with prospetive users; informal feedback from pilot trials; direct observations of use; rapid clinic ethnography	Improve fit with recipients; to address cultural factors; increase satisfaction of implementer; increase satisfaction of recipients; improve fit with implementers	content, feature	(+) improves ability to refer lower-income patients to useful and feasible resources

(+) represents the adaptation being a positive equity implication, (−) represents the adaptation being a negative equity implication and (0) represents uknown/unlikely impact on equity.

Several adaptations were made to increase the patient-centeredness and personalization of the PREVENT tool. Based on interviews with care team members and input from our advisory board, we increased the interactivity of the goal setting allowing users to edit and turn goals on and off. This change, in combination with the added element that displayed patient's motivation to change their behaviors, allows the care team member to better work with the patient to provide goals that are a better fit for them, ultimately increasing the satisfaction of the recipient. Based on feedback and direct observation from our pilot trials, we made several adaptations to improve the intervention. First, we adapted patient demographic information to include gender identity. Second, we adapted the tailoring of food goals. Previously, patients received generic recommendations for each food behavior (e.g., vegetable intake, whole grain consumption) that were not being met by the patient. Now goals are personalized based on the patients current behavior. For Instance, if a patient is currently eating 3 servings of vegetables per week, their first goal would be to eat 4 servings of vegetables per week. Over time, these goals will gradually increase or decrease the consumption to meet the overall recommendation.

Several changes were focused on increasing patient engagement with the PREVENT tool and its content with the goal of increasing the usability of the tool, improving fit with recipients, and increasing retention. The prescription that is delivered to the patient following the clinical encounter was improved by improving the literacy level, adding more information on resources that were selected, updating graphics, enlarging text, and adding more educational material. While these changes have many positive potential implications for equity, the content is not available in a language other than English and may still not be understandable for illiterate populations. Physical activity and food intake behaviors were moved to the top of the risk profile to make them the first thing patients see when viewing the tool. Pilot trials of PREVENT had low response rate to monthly follow-up surveys that examine patient's attainment of their physical activity and food intake goals. To increase engagement and retention, the length of the survey was reduced, and language was amended for clarity. The responses to these surveys are displayed in PREVENT; the display was changed to a visual chart that makes it easier for care team members to track patient progress. Additionally, the barriers to achieving their goals reported by the patient are displayed in the tool to help care team member's better support their patients.

**Figure 3:**
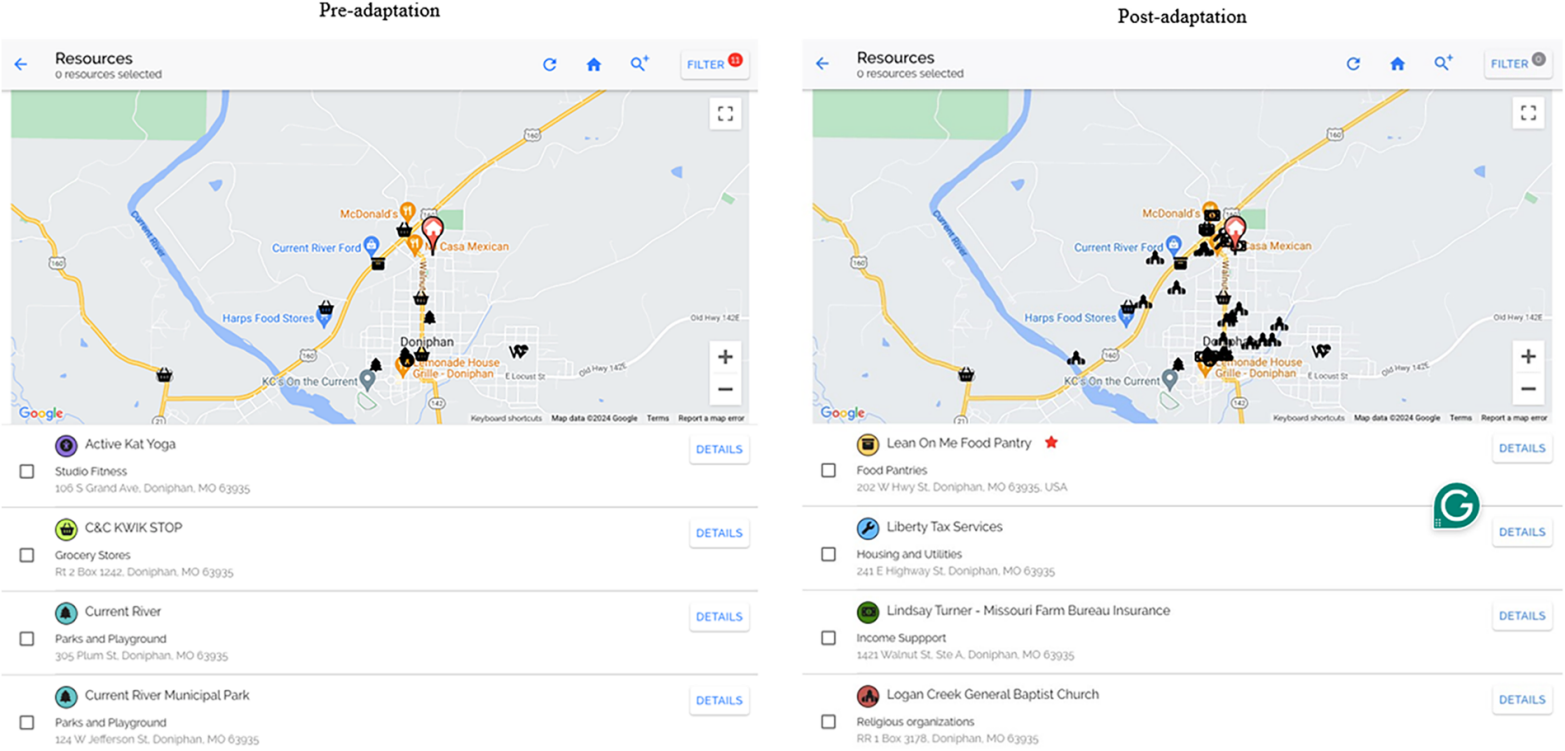
Side by side of research map. The post-adaptation resource map was expanded to resources that support social needs more broadly. The post adaptation includes functionality for care team members to add/edit resources, making them verified resources, * = the resource is active and has been verified by team member.

Our pilot trials were conducted in urban areas but did include 29% rural patients, who received fewer community resources (*n* = 1.5) than those patients in urban areas (*n* = 5.4). To improve the functionality of the PREVENT resource map for rural communities, we made several adaptations based on learnings from interviews with rural patients and care team members, direct observation and rapid clinical ethnography of rural clinics, and informal feedback (Figure 3). For this adaptation, we relied heavily on input from rural care team members and clinics. To improve the fit with implementers and their limited clinic time, we adapted PREVENT to ask patients which resources would be most helpful to them and use that information to automatically select five preferred resources within a half-mile radius of their home. If five resources are not available within a half-mile radius, it will expand until there are five available resources that meet the patients' preferences and needs. We also added a digital and remote resource library that hosts websites, apps, national hotlines, etc. to improve fit with rural communities that often do not have as many local resources.

For both our resource map and digital and remote resource library, we expanded the resources from only those specific to physical activity and nutrition to resources that support social needs more broadly, including clothing and household items, education, employment, housing and utilities, income support, individual and family support, and transportation resources. We also adapted the information that was provided for each resource to include cost, eligibility criteria, and services provided. This allowed us to include a feature in the tool that allows users to filter resources to find those that are free of charge. As available resources are constantly changing, especially in rural communities (39), the tool allows care team members to manually update resources. We added the date the resource was last updated and a verified check mark that displays on the map for resources that were manually entered. Community health workers in rural clinics were key drivers of these changes, as they felt this would increase their trust in the tool and allow them to share resources more easily across their network of community health workers.

Interviews with care team members who implemented PREVENT and direct observations during pilot trials elucidated changes that could be made to PREVENT to improve its fit with the care team members and clinic workflow. The risk profile allows care team members to move slider bars to interactively show how changes in their cardiovascular health factors (e.g., physical activity, food intake, BMI) impact their overall cardiovascular health. So that providers do not mistakenly change the patient's data, the tool was adapted to default to a simulation mode. To change the patients’ cardiovascular health data, the provider is now able to toggle to a patient data mode. In our pilot trials, multiple care team members delivered PREVENT to patients. Updates were made to the refresh capability to allow for real-time updates between simultaneous users of a single patient profile. These changes will increase the feasibility of using PREVENT as a team, usability, and satisfaction of care team members (implementers).

### 3.1 Potential equity implications of adaptations to the PREVENT tool

Expanding the tool for patients 18+ years of age increases the reach of potential benefits to all populations. Updating to align with the AHA's Life's Essential 8 adds sleep behaviors to the tool which allows providers to address this behavior among minoritized racial/ethnic groups and low-SES populations who have higher prevalence of sleep deficiency. Several adaptations (i.e., ability for care team to edit the goal, displaying patient's motivation to change their behaviors, automated tailoring of food goals to patient's current behavior) improve the ability of providers to tailor goals for physical activity and healthy food intake to meet the needs and preferences of the patients. However, this leaves room for providers to bias the goals they received (e.g., not delivering goals based on the belief a patient cannot achieve it). The tool now includes patient's gender identity to allow providers to use patient-centered language which may improve rapport/trust. However, this information could potentially allow providers to have biases related to gender identity toward that patient. Furthermore, some patients may be offended when asked about their gender identity. All content delivered to the patient was improved by increasing font size to accommodate all readers, ensuring the literacy level was understandable for patients, adding educational materials for those less knowledgeable about health behaviors and cardiovascular health and including more information on resources for patients who are not able to access the internet to look up information on their own. To reduce bias and decrease the focus on weight, BMI was moved from the top of the risk profile and replaced by physical activity and food intake behaviors to further emphasize their importance.

Adaptations to resources will increase the resource allocation and equitable availability for all patients, regardless of their ability to pay and where they live. Social needs are disproportionate among historically marginalized populations, including rural communities, and have an impact on patients' health behaviors and outcomes (6–12). The addition of social needs resources aligns with the Chronic Care Model (13, 40–42), which recognizes that quality care for obesity should connect patients with health-promoting resources, such as healthy lifestyle programs, social services, and food resources, to generate meaningful and equitable change across populations and especially in rural communities that face unique barriers (13, 43–46). The tool automatically identifies preferred resources that are close to the patient's home (when available) to increase access, particularly for those without reliable transportation (47). Some communities, particularly rural areas, have fewer resources available which may limit the ability to deliver resources close by ultimately, reducing the fidelity of the intervention to these patients. However, the care team may tailor resource delivery to provide those near their workplace, school or other commonly visited areas to improve access. Additionally, the digital resource library was expanded to provide more options for those with limited access to resources. Digital resources may also increase access to free or low-cost resources and do not require travel. While digital resources may benefit some patients, those without internet access or with low digital literacy may not be able to benefit from these offerings. The centralized source of resources that can be shared across the care team may increase the efficiency of care team members (e.g., community health workers, CHWs) in delivering resources, ultimately expanding their ability to reach a larger number of patients.

## 4 Discussion

Our study engaged end-users to adapt the PREVENT digital health tool to integrate social needs resources to improve health behavior counseling for rural adults with overweight and obesity. With intentional consideration of unique needs of rural communities. Adaptations were informed by learnings from two pilot feasibility trials and a planning grant that used interviews with care teams and patients, direct clinic observations, advisory boards, and engagement with rural community organizations. Engaging partners to proactively adapt our digital health tool was critical to meet the unique needs of adult patients and clinical care settings in the rural context and increase the likelihood of generating the intended impact among rural, low income, adult patients. Based on feedback from partners and future users, we included information about resource costs, eligibility for using the resource, and options for obtaining financial assistance. It's important to note that the tool does not create new resources; instead, it was designed to work within existing resource capacities, with a deliberate focus on cost and affordability as key factors in access. This project was in preparation for a clinic-randomized trial of the PREVENT tool in rural federally qualified health centers to examine the implementation and effectiveness on the quality of health behavior counseling, patient behaviors and their cardiovascular health outcomes.

Utilizing the FRAME systematic tracking process supported the intentionality and transparency of adaptations, ensured adaptations were supportive of equitable care and outcomes for patients, and prepared us for future implementation. Future testing will allow us to understand how and to what degree the adaptation affected implementation and the effectiveness of our tool (26, 27). Our study is one of the few studies to engage users and systematically track adaptations to a digital health intervention (48). Our study provides additions and modifications to FRAME that not only may allow better tracking for adaptation of digital health tools, specifically, but also may provide tracking of end-user participation in adaptations and equity implications of adaptations that may be useful for a variety of intervention types and settings. Further testing of these changes may be necessary to ensure that modification for digital health is comprehensive and clear for future use in digital health adaptation tracking.

The adapted PREVENT tool expands reach to all patients, aligns with evidence-based AHA guidance and increases the patient-centeredness, patient engagement, and alignment with workflow. Using a patient-centered and patient-engaged approach to behavior counseling aligns with the Chronic Care Model for obesity care (43). Health behavior counseling can be of poor quality and has shown mixed effectiveness due to educational barriers and lack of resources. Information about patients' current behaviors and challenges they face in their environment is not assessed routinely or communicated meaningfully (e.g., using visualization) to healthcare teams to tailor discussions (49–52). An informed, activated patient is vital in the Chronic Care Model; engaging patients in their care supports patient autonomy and self-determination, promotes confidence and trust in the clinician-patient relationship, and improves satisfaction with care (43, 53, 54). Adaptations to our tool also support the inclusion of multiple healthcare team members (e.g., CHWs) to engage patients and integrate community resources to address patients' social needs and improve care quality (13, 55). This partnering approach is more likely to result in behavior change by eliciting the patient perspective, addressing unmet social needs that hinder ability to adopt cardiovascular health behavior changes, adapting to resistance, and increasing motivation for tailored goals (22).

While this study had many strengths of engaging diverse implementers and partners using a variety of methods, our limited sample size may limit generalizability. We acknowledge while adaptations were made to fit the context of rural southeast Missouri and our clinical partner, these adaptations may not be generalizable to all rural communities and federally qualified health centers. For example, our clinical partners have resources such as an electronic health record system and CHWs embedded in their clinics that may not be present in all clinical care settings. While adaptations were made to improve the reach to all rural patients regardless of access to internet, changes may not completely overcome the structural challenges of limited and unreliable broadband access for some patients. Testing will need to examine whether the fidelity and effectiveness of the intervention is maintained for rural patients without internet access. It is important to note that while the coding of potential equity implications was conducted in a systematic group process, informed by conceptual underpinnings from the health equity literature, the ratings are subjective. We did not measure these equity impacts in our previous trials of PREVENT. The purpose of this coding is to generate hypotheses about equity implications and specific indicators that we can evaluate and report on in future trials.

Design considerations in which the needs of the end user are central to the intervention development have been suggested to increase the relevance and effectiveness of digital interventions as well as other types of interventions. This study used multiple user-focused methodologies to improve the fit of the PREVENT tool for rural communities and clinics. The PREVENT tool has the potential to improve the quality of health behavior counseling and promote behavior change to reduce obesity and CVD risk among a high-risk population. Future researchers undertaking adaptation work should be aware of tools like Design for Dissemination (D4D), which facilitate collaboration with partners and help track decisions being made. Ongoing and future trials will seek to test the implementation and effectiveness of the PREVENT intervention, and determine additional adaptation needs for specific settings and patient populations. This paper offers an approach and coding tool other teams can use to document and report adaptations made to digital health tools to improve the specificity of adaptation tracking and transparency of reporting.

## Data Availability

The original contributions presented in the study are included in the article/Supplementary Material, further inquiries can be directed to the corresponding author.
