# Supplementary material for: Adaptation of a digital health intervention for rural adults: application of the Framework for Reporting Adaptations and Modifications-Enhanced
Source: Front Digit Health. 2025 Feb 18;7:1493814. doi: 10.3389/fdgth.2025.1493814 (PMC11876167; doi:10.3389/fdgth.2025.1493814)
Supplement: Supplementary file 1 [file Table1.pdf]

Supplementary Table 1: Codebook

Column Name	Column description	Response options	Response definitions
Description change	description of change made	free text	No further definition
Data source/method informing the change	method by which information or suggestions informing the change was collected or obtained (formal or informal)	interviews w/ users	data collection during PREVENT pilot study w/ implementing practitioners
		interviews w/ prospective users	interviews with potential future PREVENT users (e.g., CHWs, dietitians at Highlands)
		Direct observation of use	formal observations during PREVENT pilot to see use in the clinic
		Rapid clinic ethnography	clinic visits to observe workflow with potential future users
		informal conversations during pilot trials	conversations with implementing practitioners (not formal data collection)
		advisory board	feedback from advisory board members
When did modifications occur?	what time period related to routine integration of the tool	pre-implementation/ planning/pilot	before pilot PREVENT trials
		implementation	during pilot PREVENT trials
		scale-out	rural planning project
		scale-up	No further definition
		maintenance/ sustainment	No further definition
Were adaptations planned?	whether changes were intentional or unintentional	planned/proactive	intentional change made in anticipation of a need
		planned/reactive	intentional change made in response to a need, barrier, or suggestion

		unplanned	unintended change, likely made by implementer in the course of delivering the PREVENT intervention
By WHOM were modifications made?	individual(s) who made the decision to implement the change + the individual(s) who enacted the change (e.g., programmers who built it into the tool)	intervention developer	principal investigator
		software developer	software developer
		researcher/ research team	principal investigator, co-investigators, research assistants, project manager
		implementing clinicians	clinicians who delivered PREVENT during pilot trials
		prospective users	care team members (e.g., physician + dietitian + CHW + embedded research coordinators) who are potential future PREVENT users (implementers)
		recipients	patients/caregivers who receive PREVENT
		community members	individuals/groups outside the clinic or research team (e.g., patients on advisory board, CRC)
		political leaders	No further definition
		program leader	No further definition
		funder	grant funding agency or program officer
		program manager	clinic managers/ supervisors who make decisions about tools use in their clinics
		professional organizations	e.g., American Heart Association
		administrator	e.g., health system CEO or upper level leadership

Who participated in decision?	source of the recommended change or who contributed to decision other than who made change	same options as by whom modifications are made	e.g., suggestion came up in an interview, recommendation from advisory board
What was the goal?	What was the purpose of making the change, what is trying to be achieved	reach	increase # of patient recipients
		retention	keeping patients in the intervention (e.g., doing goal check-in surveys)
		feasibility	intervention easy to integrate into the clinic; workflow/doable in clinic setting
		increase usability	improve ease of use/user friendliness of the tool itself (how easy tools' features & functionality are to navigate, not about content)
		improve fit with implementers	improve the appropriateness of the intervention content (tool + delivery workflow) with the care teams
		improve fit with recipients	improve the appropriateness of the tool content with patients (usefulness)
		to address cultural factors	social context patient population (e.g., rurality, literacy levels, recommendations align with social or familial practices)
		improve effectiveness/ outcomes	enhance care quality, improve health behaviors & CVH
		increase satisfaction of implementer	make tool more likable to care team

		increase satisfaction of recipients	make tool more likable to patients & caregivers
		reduce cost	decrease cost of clinic operations or tool maintenance
		increase interaction	increase use of the tools features (e.g., simulating changes with slider bars, tailoring goals to patient need)
What is modified?	what kind of change is made to the tool, the process of using it, or how it is integrated into the clinic workflow	content	modifications made to the content within the tool (e.g., health info, goals, cut points)
		feature	aspects of the tool that determine its function (e.g., slider bars, text entry boxes, organization of displays, color coding, icons, fonts, etc.)
		context	workflow surrounding the tool (e.g., care team members who deliver, patient populations to whom PREVENT is delivered, type of setting)
		training & evaluation	changes made to training materials, how training is conducted
		implementation strategies	changes made to the strategy package used to support integration, fidelity, & sustained use of PREVENT
At what LEVEL OF DELIVERY (for whom/what are modifications made?)	target groups or setting level for which changes are made	target patient group	changes made to PREVENT or how it is delivered to benefit/apply to any eligible patients broadly (i.e., anyone with overweight/obesity)

		cohort that share a characteristic	changes made to PREVENT or how it is delivered to benefit a particular subset of patients (e.g., age group, rural, health condition, income level, literacy levels)
		individual patient	No further definition
		practitioner level	changes made to PREVENT or workflow to meet needs of a particular type of clinician (e.g., physician)
		clinic/unit level	changes made to PREVENT or workflow to meet needs of a group of providers in a clinic
		organization	changes made to PREVENT or workflow to meet needs of a group of clinic practices or organization administration
		network system/ community	changes made to PREVENT or how it is delivered to benefit groups in + outside the clinic (e.g., community resource council)
Contextual modifications are made to which of the following?	type of changes made to the conditions surrounding use of the tool (not PREVENT tool itself)	format	PREVENT data input or delivery modality (e.g., printing prescription instead of electronic delivery)
		setting	type of location in which PREVENT is delivered (e.g., type of clinical practice, non-clinical location)
		personnel	who delivers PREVENT or is involved in the workflow (e.g., adding CHWs)
		population	patient population with whom PREVENT is used (e.g., extending age group, change in BMI eligibility)

What is the NATURE of the content modification?	classification of the type of change made to the PREVENT tool itself or the workflow for delivering it	tailoring/tweaking/ refining	minor changes made to meet the needs of individuals, not a widespread change
		changes in packaging or materials	modifications to data collection instruments (e.g., adding sleep items to survey), how info is packed/displayed in the tool (e.g., risk profile display), materials sent to patient (e.g., prescription)
		adding elements	new info or tool content (e.g., digital resource list)
		removing/skipping elements	taking content out, skipping a step in tool delivery (e.g., no review of risk profile)
		shortening/ condensing	reducing the pace or timing of PREVENT use/delivery
		lengthening/ extending	increasing the pace or timing of PREVENT use/delivery (e.g., repeat use w/ patients)
		substituting	swapping content (e.g., dietitians use different food list)
		reordering of intervention modules/ segments	changing the order in which PREVENT is presented (e.g., starting w/ goals rather than risk profile)
		spreading	breaking up content over multiple sessions (e.g., risk profile, goals reviewed in 2 separate encounters)
		integrating parts of the intervention into another framework (integrating)	e.g., selecting elements of PREVENT to deliver with another intervention

		integrating another treatment into the EBP	not using the whole protocol and integrating other techniques or interventions into the delivery of PREVENT, (e.g., PREVENT + CBT for weight management)
		repeating elements or modules	No further definition
		loosening structure	No further definition
		departing from the interventions followed by a return to the protocol within the encounter	No further definition
		drift from protocol w/o returning	No further definition
What is the nature of the feature modification?	classification of the features of the tool	changes in interface	changes to visualizations to improve visual appeal, aesthetic
		changes in interactivity	e.g., slider bars, buttons, text boxes; filtering
		changes in function	back-end improvements (e.g., improving speed of resource map, level of zoom in/out, streamlining for simultaneous users)
		digital accessibility	changes to make the tool more accessible for different literacy levels or abilities (e.g., using images/icons vs. text, changing font size, simplifying text, use on various device types)
Relationship fidelity/core elements	whether change preserves core	fidelity consistent	core components/functions preserved

	components of PREVENT intervention	fidelity inconsistent	core components/functions not preserved, are changed
		unknown	No further definition
Potential equity implications	describe whether/how change is made with the intention of promoting equity	(+) potential to enhance equity	No further definition
		(-) potential to diminish equity	No further definition
		(0) unlikely or unknown impact on equity	No further definition
Reasons: Sociopolitical	current or historical social, political, economic factors broadly in society that may influence health system or patient circumstances that inform PREVENT adaptations or that changes try to respond to	existing laws	No further definition
		existing mandates	federal or state mandates that set care standards (e.g., requirements for weight counseling with patients who have high BMI)
		existing policies	health policies e.g., ACA
		existing regulations	regulations for how care is delivered (E.g., provider licensing & scope of work)
		political climate	No further definition
		funding policies	public or private payer policies (e.g., Medicaid or HRSA quality metrics to obtain funding)
		historical context	local, regional, or national historical context that may shape current trends, needs (E.g., racial segregation, redlining)
		societal/ cultural norms	social or cultural norms, values, beliefs (e.g., medical mistrust, racism, discrimination)
		funding or resource	state, federal, or local funds or resources for healthcare, health

		allocation/availability	promotion, community resources, etc.
		updated guidance	e.g., new research evidence, changes to professional guidelines, practice guidelines, etc. (E.g., AHA updates to essential 8)
Reasons: Organization/ Setting	change driven by or addresses a determinant at the organization or implementing inner setting (e.g., clinic) level	available resources	personnel, space, financial, physical or digital resource to support PREVENT's integration & sustained use
		competing demands or mandates	organizational priorities or needs that may align or compete with PREVENT
		time constraints	limited time in clinical encounter
		service structure	how care is organized in the clinic setting
		location/accessibility	where care is offered (e.g., clinic location, telehealth)
		regulatory/ compliance	Organizational rules, policies, practices
		billing constraints	how care delivery, health services are coded for, billed (e.g., billing for physician encounter vs. CHW)
		organization	organization culture, climate, leadership support
		mission	goals, values of the organization (e.g., FQHC vs. for profit health system may have different mission)
		cultural or religious norms	social demographics of the organization (e.g., care team members from rural area who have close ties to patients)

Reasons: Provider	provider or care team member characteristics, attributes that influence PREVENT adaptation or features that adaptation tries to respond to	race/ethnicity	No further definition
		sexual/gender identity	No further definition
		first/spoken language	No further definition
		previous training and skills	No further definition
		preferences	something liked, favored by provider(s) or care team member(s)
		clinical judgment	care decisions made by providers
		cultural norms, competency	provider cultural competency, care team group norms, social norms among professional roles
		perception of intervention	care team beliefs, attitudes towards PREVENT, health behavior change intervention
Reasons: Recipient	patient & family characteristics, attributes, social determinants of health, or lived experience that influence adaptation or that adaptation tries to respond to	race/ethnicity	No further definition
		gender identity	No further definition
		sexual orientation	No further definition
		first/spoken language	No further definition
		literacy/ education level	No further definition
		cognitive capacity	No further definition
		physical capacity	No further definition
		comorbidity/ multi-morbidity	No further definition
		cultural or religious norms	No further definition
		legal status	No further definition
		immigration status	No further definition
		crisis or emergent circumstances	No further definition

		motivation & readiness	No further definition
		access to resources	No further definition

* Blue text= modification to existing content; Green text= new domain/construct
