# Supplementary material for: Adaptation of a digital health intervention for rural adults: application of the Framework for Reporting Adaptations and Modifications-Enhanced
Source: Front Digit Health. 2025 Feb 18;7:1493814. doi: 10.3389/fdgth.2025.1493814 (PMC11876167; doi:10.3389/fdgth.2025.1493814)
Supplement: Supplementary file 2 [file Datasheet1.pdf]

Prospective User (Healthcare Team Member) Interview Guide

Introduction (5 minutes)

[Interviewer note: PCP indicates items to ask of primary care providers (e.g., physicians, PAs, nurse practitioners) only]

Stakeholder Discovery (10 minutes)

Let's get started. The first portion of this interview will be general questions about your role, your workflow and the challenges you face in treating patients with overweight or obesity.

1. Can you tell me a little bit about your practice?
    a. Setting of care?
    b. Type of patients you see (age distribution)?
    c. What proportion you would say meet the clinical definition of obese? Overweight?
    d. Length of typical patient encounter?

2. What is your role in helping overweight or obese adolescents adopt healthy behaviors?
    a. How comfortable do you feel in talking to patients about their weight? About adopting healthy behaviors?

3. What healthcare team member do you think is best suited to deliver behavior change goals to patients with overweight and obesity? (PCP)

4. When you encounter a patient who is overweight or obese, what are the main things you are trying to achieve? What are you required to do? (PCP)
    a. What does success mean to you?
    b. What metrics do you use to measure success?

5. What are some of the main challenges your patients face to eating healthy or being physically active?
    a. What kinds of resources do your patients need to support healthy habits? (examples: access to affordable produce and other healthy foods, access to safe spaces to be physically active)

User Testing (15 minutes)

Now I am going to have you use the tool we developed for providers, like you, to use with your patients who are overweight or obese to prevent the development of cardiovascular disease.

Send link and log-in credentials in the zoom chat. This interaction should be the first interaction with the tool and we want to avoid the user using the tool and becoming familiar with it before "the interaction." Once the provider has logged into the tool- ask that they share their screen.

Click the link I just sent you in the chat box and login using the credentials. Click on the patient "Interview Patient." Then click on the box below the patient information that says "preparing" and the date. We would appreciate if you could share your screen so we can view what you are doing.

We realize this is your first time seeing the tool but our goal is to understand how you would engage with this tool to identify problems with the design and hear your thoughts on how this would fit in your workflow. We will do this by having you think aloud as you use the tool. We envision a healthcare team member and a patient viewing this tool together. You can talk to me as if I am your patient (a 40-year old female with obesity) or simply talk through your impressions of the tool. We will orient you to each page of the tool, allow you to interact and give your initial thoughts and then prompt you with specific questions. Any questions before we get started?

Great let's get started. You are within your patient profile that includes their most recent health data, which is used to calculate an overall cardiovascular health score based on the American Heart Association's Life's Simple 7 behavioral and clinical factors. This health score and each health indicator are categorized into poor, intermediate, or ideal ranges based on standardized cut-offs. Now I'll give you a minute to review this first screen. When you're ready, please give your impressions and feel free to think out loud to verbalize any thoughts or questions that come to mind as you review this.

Prompts:
- What do you think as you look at this screen?
- How easy or difficult would it be for you to navigate this page during a patient encounter?
- How does the tool organization look to you?
- What are you thinking about the tool? What do you like/dislike?
- What decision are you trying to make?
- Who in the care team do you think would use this with patients?
- What about this would your patients find useful or meaningful?

When you are ready to move on, I will have you select the "prescription" tab from the bottom of the page. This page includes physical activity and nutrition recommendations based on the patient's current behaviors and health status. The page also has an interactive community resource map and a digital resource repository. Please review this page and when you are ready, share any thoughts or questions that come to mind. [*Repeat prompts from above]*

Community Health Worker-specific prompts: Can you tell me about your experience with:
- Tailoring physical activity goals
- Toggling between food intake recommendations
- Using the resource map to locate and select resources
- Reviewing and selecting digital resources
- Sending an electronic prescription

Follow-up Questions (10 minutes):

1. What barriers do you foresee in using this with patients?
    a. What would help overcome those barriers?

2. How do you think this would fit into your workflow for a typical patient encounter?
    a. [if it wouldn't fit]: Can you describe why this might not be a good fit? What are some ways to improve how this fits into your workflow?

3. Would you like this to be integrated into the EHR?
    a. If so, let's think about how this could look inside the EHR…
        i. how would you like to be alerted that a patient is eligible for this?
        ii. How would you like to access PREVENT from the EHR? (e.g., side panel, new tab, embedded link to external web page)
        iii. How would you like to view the information from the PREVENT tool?
    b. If not, why not? Would you prefer partial integration of some components or information? (e.g., PREVENT prescriptions sync with patient encounter)

4. Who do you think should use this tool with patients?
    a. How might a team-based approach to using PREVENT look in your clinic?
    b. How would you feel about CHWs delivering behavior change goals and resources? (PCP)

5. What type of training do you think healthcare team members would need to effectively use the tool?
    a. How would you like to receive this training (e.g., via asynchronous videos, live online training, in-person)?
    b. How useful would training or resources on motivational interviewing or shared-decision making be?

6. Besides training, what other supports or resources would help healthcare team members use PREVENT (e.g., user manual, quick tips sheet, practice sessions with research team)?

Follow-up Questions (5 minutes):

To wrap up, we have a few brief closed-ended questions about your impressions of the tool.

Launch the zoom poll which will ask participants to rate the following on a 5-point likert scale (strongly disagree to strongly agree):

1. The information the tool provides is useful.
2. The information is presented in a useful format.
3. The tool was easy to use.
4. The tool seems possible to use with my patients.
5. The tool would help me be more effective.
6. The tool would make the information I want easier to access.
7. The tool would help meet my needs when providing care for overweight or obese patients.

**If time, probe on responses that indicate strongly disagree or disagree to understand why.*

1. Can you think of someone else I should talk with to gain insight on the tool?
    a. Perhaps someone who makes decisions about what interventions/technology to use in your practice? Or who would be likely to use the tool with patients.
